# A systematic review of qualitative studies of adults’ experiences of being assessed for psychological therapies

**DOI:** 10.1111/hex.12844

**Published:** 2019-01-08

**Authors:** Angela Sweeney, Sarah Clement, Kate Gribble, Elizabeth Jackson, Sarah Carr, Jocelyn Catty, Steve Gillard

**Affiliations:** ^1^ Population Health Research Institute St Georges University of London London UK; ^2^ Freelance Researcher London UK; ^3^ Child, Community and Educational Psychology Exeter University Exeter UK; ^4^ SGUL Library St Georges University of London London UK; ^5^ School of Social Policy University of Birmingham Birmingham UK; ^6^ Department of Education and Training Tavistock and Portman NHS Foundation Trust London UK

**Keywords:** CBT, client experience, client involvement, counselling, IAPT, psychological therapy assessments, psychotherapy, qualitative research, service user experience, service user involvement, systematic review, thematic synthesis

## Abstract

**Objective:**

To synthesize the qualitative literature on adults’ experiences of psychological therapy assessments. The review was led by people with experience of undergoing assessments, with high levels of client involvement throughout.

**Search strategy:**

A comprehensive search of electronic databases was undertaken, with additional search strategies employed to locate further literature.

**Inclusion criteria:**

Studies were included that qualitatively explored the experiences of people aged 16+ who had been assessed for psychological therapy services. Assessments could be structured or unstructured. Qualitative was defined as any analysed account of people's experiences, including qualitative survey data.

**Data extraction and synthesis:**

Literature quality was appraised using the Critical Appraisal Skills Program checklist, modified to include client involvement and intersectionalities. Following data extraction, thematic synthesis was used to synthesize findings across studies.

**Results:**

Of 12 743 titles were screened, with 13 studies relevant to the review. Themes and subthemes were identified at three stages of the assessment process: the journey to the assessment, at the assessment, and after the assessment. Findings highlighted the emotional impact of assessments, collaboration, intersectionalities, rights, pathologization, socioeconomic restrictions, and information and support needs. Implications and limitations were indicated.

**Discussion and conclusions:**

Findings were situated within the trauma‐informed (TIA) literature. Trauma‐informed assessment principles, including collaborative assessments, may be fruitful means of improving people's experiences. Whilst the benefits of collaboration appear self‐evident, explicitly collaborative approaches were not the norm, nor were studies conducted independently. Further service user research is needed. A greater understanding of the experience of minority groups is also needed.

## 1 INTRODUCTION

In England, large numbers of adults access psychological therapies, with the use of such therapies having risen steadily since 2000.^1^ Nearly one million people are assessed annually in England through the National Health Service (NHS) Improving Access to Psychological Therapies (IAPT) programme alone^2^ (IAPT is an English NHS therapy programme delivered through local services and free at the point of delivery). This figure is set to rise to 1.5 million by 2020.^3^ This indicates that huge numbers of people in England are assessed for psychological therapies every year, with an upward trajectory. Note that we are using the term “psychological therapies” to refer to various talk‐based therapies including counselling, psychotherapies, cognitive behavioural therapy.

Prior to delivering a psychological therapy, an assessment is undertaken during which assessors establish service suitability and eligibility, considering whether and how psychological therapy might help. Approaches vary from unstructured history gathering or storytelling methods^4^ to structured assessments of symptoms,^5^ or some combination of both. Assessments can take place many months before therapy begins or seamlessly lead into therapy. Whilst people within IAPT typically receive a single brief telephone assessment, beyond IAPT, assessments can be one‐off information gathering exercises or multiple sessions which aim to have therapeutic impact.^6^

The ways in which practitioners conduct assessments are influenced by factors such as service cultures; bureaucratic requirements; personal skills and qualities; early training; theoretical orientation; practical experience; client factors; therapeutic alliance; and how these meld within specific assessments.^7^, ^8^ Thus, whilst there are broad approaches to assessment, individual encounters are inevitably intuitive, idiosyncratic and vary from assessor to assessor and encounter to encounter.^8^

There is some evidence that psychological therapies can cause long‐term harm^9^ and that people who identify as LGBT and/or as black and minority ethnic are more likely to report harms.^10^ Hardy and colleagues found that a clear assessment, amongst other factors, fostered engagement and helped mitigate against potential long‐term harms.^11^

There is a growing international literature on trauma‐informed approaches (TIAs). Such approaches understand the role of violence and trauma in the lives of many who seek psychological support; ensure that systems and practitioners are sensitized to this and do not (re)traumatize; and are strengths based, understanding that people are attempting to survive.^12^ Trauma‐informed assessments aim to be informed by cultural, religious, gender, language, socioeconomic, age and disability awareness; focus on therapeutic alliance and collaboration; and have clear and transparent processes.^13^ Assessors ensure questions are necessary, make sensitive and carefully timed and paced trauma enquiries, do not ask for trauma details, support grounding and focus on immediate safety.^13^, ^14^, ^15^, ^16^ This TIA literature will be used to inform an understanding of people's experiences of psychological assessments.

Despite an established culture of service user involvement in mental health, psychological therapies lack almost any client involvement. Developing an involvement culture could enable services to enhance ethical practice, minimize harms and reduce dropouts.^17^ However, Trivedi argues that psychological therapies are often resistant to client involvement, for instance, arguing that feedback is “transference,” that people have an “axe to grind,” are too vulnerable to be involved or are unable to comment dispassionately.^17^ We will consider client involvement levels in our review papers.

The purpose of this review was to synthesize qualitative research exploring adults’ experiences of undergoing psychological therapy assessments to develop a rich and comprehensive understanding that increases best practice knowledge. The review is being conducted as part of a wider study investigating assessment processes for talking therapies (APTT).

## 2 METHODS

This study had four main phases: (a) formulation of the protocol; (b) systematic searching and selection of literature; (c) data extraction and quality assessment; and (d) data synthesis.

### 2.1 Formulating the protocol

A Service User Advisory Group (SUAG) established the review priorities including focus, literature types and key terms. A draft protocol was discussed and revised in a SUAG subgroup. The SUAG, a Clinician Advisory Group and a research librarian, reviewed the protocol, leading to revisions. Search terms were further revised following piloting.

#### 2.1.1 Inclusion and exclusion criteria

Studies that met the following criteria were included:

- *Population*: adults aged 16+ who have been assessed for a psychological therapy service for their mental health.
- *Phenomena*: explores an aspect of people's experiences of being assessed using structured or unstructured approaches. Assessment was defined as a process.
- *Study type*: any analysed account of people's experiences, including qualitative components of broader studies and qualitative survey data.

Exclusion criteria were (a) people aged 15 or under; (b) studies where it was not possible to disaggregate clients’ and other's views; (c) social or cognitive assessments; (d) conference proceedings/abstracts; and (e) publications not in English. There were no restrictions by date or setting.

### 2.2 Systematic searching and selection of literature

#### 2.2.1 Search strategy

Four electronic databases were searched in January 2015, updated on August 2017: CINAHL (Cumulative Index to Nursing and Allied Health Literature), EMBASE, PsycINFO and MEDLINE. Search terms were applied using free text and subject headings (see Table S1 for final search strategy and Table S2 for an example).

In consultation with Advisory Groups, additional literature searching included (a) WorldCat Dissertation and Theses, and OpenGrey, searched in February 2015, updated in August 2017; (b) a call for literature was placed in a national service user/survivor organization newsletter; (c) forward and backward citation tracking of included papers and some relevant excluded papers; (d) four journal indexes were searched: Psychological Assessment; Journal of Counseling Psychology; International Journal of Mental Health; and Psychology and Psychotherapy: Theory, Research and Practice; (e) where possible, lead authors of included papers were asked for relevant literature; and (f) requests to advisory groups and key experts. This enabled us to identify literature beyond peer‐reviewed journals.^18^

#### 2.2.2 Screening and selection of studies

Two authors screened retrieved records from the 2015 peer‐reviewed database searches for potential inclusion KG and SC. Each author screened 50% of records (n = 3957 records per screener), with 4% (n = 358) of records double screened. A Kappa calculation on the results of double screening found that the strength of agreement between screeners was poor (unweighted *κ *= 0.074; 95% CI = 0‐0.469). A third screener AS reviewed the records that each screener had identified as potentially relevant. SC then rescreened all references as there was strong agreement between AS and SC had extensive experience in systematic reviews. The full texts of identified studies were assessed for eligibility by two people AS/SC. In the 2017 update search, SC screened titles/abstracts and AS and SC assessed studies for eligibility based on the full texts. Discrepancies were discussed and resolved with an arbiter SG.

For the grey literature, one author screened the retrieved titles SC, located and read full copies of texts and arrived at a list of potential titles. A second author then reviewed the full texts for their possible inclusion AS. There were no discrepancies.

### 2.3 Data extraction and quality appraisal

#### 2.3.1 Data extraction

Data extraction was discussed in a SUAG subgroup and piloted. A standard data extraction form, used by AS, extracted basic information such as country, methods, participant socio‐demographics and assessment form (see Table S3). First authors were contacted (where possible) for clarifications and to describe client involvement. Extraction of study findings is described under Data Synthesis.

#### 2.3.2 Quality appraisal

We piloted three quality appraisal approaches^19^, ^20^ including a bespoke approach used in EPPI‐Centre reviews (these build on the quality assessment frameworks of previous EPPI reviews).^21^, ^22^, ^23^, ^24^ Like Malpass and colleagues, we concluded that the CASP included a range of issues whilst remaining manageable.^25^ To ensure the review was client focused, we modified the CASP to include intersectionalities and client involvement (see Table S4).

Feder et al^26^ piloted four approaches to scoring the CASP and found a simple unweighted score was most effective, and so we also adopted this approach. Our aim was not to exclude papers based on quality as there is a lack of consensus over quality appraisal methods, and poor or limited reporting does not necessarily equate to unreliable findings.^25^ Instead, we aimed to gain some understanding of the strength of the evidence base and have reported the overall quality of the body of literature, rather than individual scores.

### 2.4 Data synthesis

Literature was analysed using thematic synthesis.^27^ First, literature was read and extensive notes were taken, with the whole text considered data. From this, a draft coding frame was created, discussed by the SUAG and applied using Microsoft Excel. The coding frame contained descriptive and analytical themes, subtheme, linkages across the data and indicative quotes and was refined and expanded as coding continued. Findings were discussed in a reflexive data workshop with SUAG members.^28^

## 3 RESULTS

### 3.1 Description of included studies

**Figure 1:**
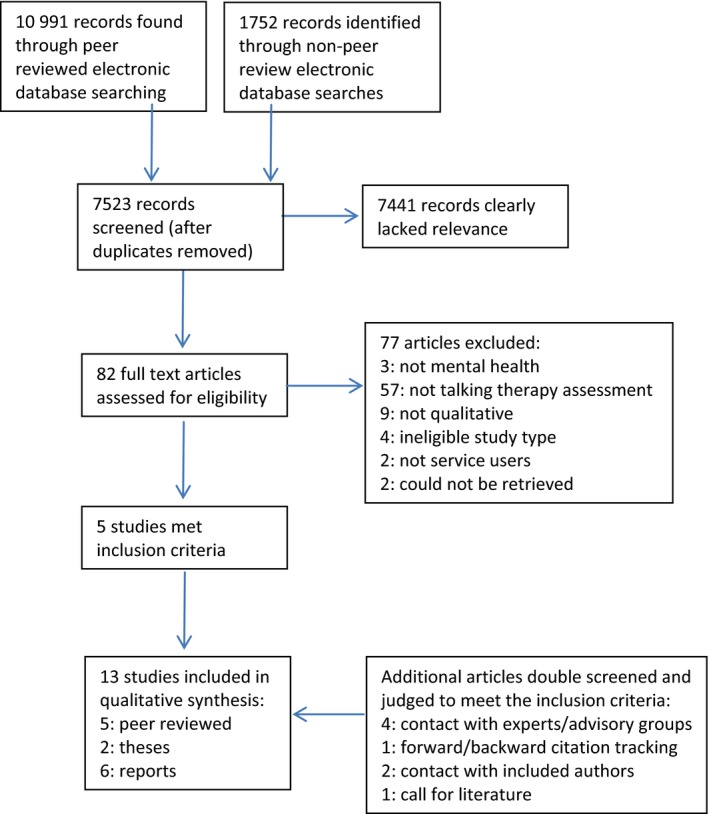
Prisma diagram showing flow of studies through the review

Searching identified 12 743 references, with 13 studies relevant to the review—see Figure 1, PRISMA diagram, for the flow of papers through the review. There were two PhD theses, six reports and five peer‐reviewed papers.

Nine studies were from the UK, with one each from the Netherlands, United States, Canada and Israel (Table 1). Five studies explored IAPT (described in the Introduction); this involves a telephone assessment followed by allocation to therapy (if eligible), typically a short course of CBT. All but one study was published in or after 2005 with six studies published in 2015‐2017. One study used a survey alone and five studies used semi‐structured interviewing alone. The remainder used interviews along with one other method (survey, focus group, audio‐recording or written account of the assessment). Ethnicity was not reported in four studies, and the majority of participants were white in all but two remaining studies. Overall, more women were included than men. Sexual orientation was described in two studies, with around half of people identifying as heterosexual in one study and around three quarters in the second.

**Table 1:** Overview of studies included in the synthesis

Lead author Year Country	Article type	Service	Assessment (purpose, assessor and procedures)	Study aims	Data collection and analysis	Participants (assesses only): numbers, gender, sexual orientation, ethnicity and age	Client involvement in the research process^a^
Barber 2017 UK	Independent report	Sutton Uplift: an IAPT, well‐being, primary care management and secondary mental health care assessment service	To establish service eligibility. The assessment could have been in any one of the four Sutton Uplift services. Procedures and assessors not described	To explore how Sutton Uplift is perceived by people who have been offered or used the service with a particular focus on access, and the support offered or received and its impact	Survey. Semi‐structured interviews. Focus groups. Co‐produced thematic‐based analysis	128 people surveyed, 29 people interviewed. Survey: most female (n = 86), white British (n = 90) and aged 36‐45 (n = 36) or 46‐55 (n = 26) (range 18+). Sexual orientation not stated	Research led by service users/clients
Bryant 2007 UK	Independent report	Statutory and non‐statutory psychological therapies in Leeds	To establish service eligibility. Procedures and assessors not described	To explore service users views about accessing psychological therapy services in Leeds, UK, with a particular focus on pathways, information, choice, and understanding and expectations of therapy	Semi‐structured interviews. Framework approach	20 participants. Roughly half female, majority white British, majority 31‐45 (n = 7) and 46‐59 (n = 6) (range 18+). Sexual orientation not stated	Research team included a service user/client researcher and service user/client consultants
Cape 2005 UK	Peer reviewed	Routine clinical assessment letters including one psychology outpatient department	Purpose of assessment unclear. 19 people assessed by a psychologist, 13 by a psychiatrist (data relating to psychologists included in synthesis). Procedures not described.	To explore people's immediate reactions to the routine clinical assessment letters sent by their psychiatrists and psychologists to their GP/other referring professionals	Semi‐structured interviews. Thematic analysis	32 participants. Half female, majority white except 2 African Caribbean and 1 mixed heritage. Age ranged from 20 to 62 (median 35). Sexual orientation not stated	None apparent
Crawford 2007 UK	Independent report	Specialist services for people diagnosed with personality disorder (data relating to two therapy services included in synthesis)	To establish service eligibility. One service used multiple meetings and forms; a second service used two meetings and a computer assessment. Assessors not described	The qualitative component aimed to explore individuals’ experiences with a particular focus on identifying factors that influence perceptions of service quality and outcomes, and decisions to engage with or withdraw from services.	Semi‐structured interviews. Focus groups. Framework analysis	108 service user participants plus 15 ex‐service users. 70% female; 71% white British, 18% white other and 11% BME. Age and sexual orientation not stated.	Qualitative study was led and conducted by service user/client researchers
Danna 2011 United States	PhD thesis	University counselling centre	Purpose not clear. Collaborative psychological assessments (or therapeutic assessment) including computer testing and feedback conducted by trainee psychologists.	To understand client and therapist experiences of collaborative assessment with in order to improve practice.	Semi‐structured interviews (including videoed extracts of the assessment). Written description of assessment experiences. Grounded theory.	5 participants. 4 male, all white, age ranged from 20 to 50 (median and mean 31).	None apparent
De Saeger 2016 The Netherlands	Peer reviewed	Clinic for people diagnosed with personality disorder	Purpose unclear. Collaborative therapeutic assessment: four sessions including psychological testing (eg, Rorschach) followed by written and face‐to‐face feedback. Assessors not described.	To elucidate and explain largely favourable process outcome results in an RCT, to generate hypotheses about the effective ingredients of therapeutic assessments from service users’ perspectives and to tighten the conceptual understanding of therapeutic assessment.	Semi‐structured interviews. Modified consensual qualitative research	10 participants. 4 female, mean age 47.3 (SD = 11). Ethnicity and sexual orientation not stated.	None apparent
Fornells‐Ambrojo 2017 UK	Peer reviewed	Routine Outcome Measures (ROMs) in IAPT‐SMI (severe mental illness) demonstration site	Purpose and assessors unclear. Use of ROMs at *baseline only* included in data synthesis.	To explore service user perceptions of Routine Outcome Measurement (ROM) focusing particularly on satisfaction and experience.	Survey. thematic analysis	257 participants. Half female, 56% from BME communities, mean age 37 (SD = 11.9, range 17‐68). Sexual orientation not stated.	None apparent
Hamilton 2011 UK	Independent report	IAPT	To establish service eligibility. Assessors and procedures unclear.	To evaluate London IAPT services with a particular focus on understanding service user satisfaction.	Survey. Semi‐structured interviews. Focus groups. Thematic analysis?	116 people surveyed, 19 focus group participants, 20 interview participants. Survey and interviews: around three quarters female, 60% white British, age range 19‐69 (survey mean age = 37; interviews mean age = 41). Sexual orientation not stated.	High levels of service user/client involvement
Hann 2015 UK	Independent report	IAPT	Varies by service, not always clear.	To understand the views of people who completed, did not engage with or discontinued therapy with a particular focus on effectiveness, satisfaction and recommendations.	Survey. Semi‐structured interviews. Thematic analysis	305 people surveyed (241 IAPT service users, 64 non‐ service users). 61 people interviewed. Survey: three quarters white and female, mean age 40 (range 16‐76). Interviews: two‐thirds female, three quarters white, mean age 41 (range 20‐76). Sexual orientation not stated.	Research team included three service user/client researchers and two mainstream researchers
Lavie‐Ajayi 2017 Israel	Peer reviewed	Psychotherapy in a community mental health centre	To establish service eligibility. Interview by a therapist/senior clinical psychologist	To explore, through discourse analysis, the tension between hegemonic and critical discourses in the clinical interaction within a single assessment encounter between a therapist and service user.	Audiotaped intake. Semi‐structured interviews. Critical discourse analysis	One female participant, Mizrahi (Jew of North African/Asian origin), aged 53. Sexual orientation not stated.	None apparent
McDonagh 1997 Canada	PhD thesis	Traditional (including psychiatric, psychoanalysis) and feminist therapy	Not clear, varies by service. Data synthesis includes data relating to psychological therapy (including psychoanalysis, psychotherapy etc.)	To explore women's subjective experiences of therapy for survivors of childhood sexual abuse (CSA).	Survey. Semi‐structured interviews. Thematic analysis?	92 women survivors of CSA surveyed, 11 women CSA survivors interviewed. Survey: half heterosexual, 90% white, median age 36 (range = 19‐58). Interviews: half heterosexual, majority white, median age 37 (range 19‐52).	Researcher identifies as having personal experience of the topic
Marshall 2016 UK	Peer reviewed	IAPT	To establish service eligibility. Assessors and procedures unclear.	To explore people's reasons for not attending therapy.	Semi‐structured interviews. Thematic analysis using data mapping sheets	14 participants, 10 female. Age range 18‐64. Ethnicity and sexual orientation not stated.	Minimal
Morris 2005 UK	Report	Individual or group psychoanalytic psychotherapy at a women's therapy centre	To assess service eligibility and whether group or individual therapy more appropriate through an interview. Assessors are therapists in the centre	To highlight women's needs, assess whether psychoanalytical psychotherapy helped them to progress in their lives and identify possible gaps in service provision with a view to improving future women's experiences.	Semi‐structured interviews. Framework analysis	47 women interviewed. 26 white British, 35 heterosexual, majority aged 30‐39 (n = 21) (range = 25‐66).	Research led by a service user/client researcher

a Clarified through email communication with study authors where possible.

### 3.2 Quality of included studies

Quality appraisal scores ranged from 5 to 11 (maximum 12) with a mean of 8. Reports tended to score higher than peer‐reviewed papers, perhaps related to journal word space restrictions.^29^ Studies were strong on the appropriateness of design (eg, recruitment), basic reporting (eg, aims) and value. Around half the studies involved service users/clients in the research process (7/13) and/or considered issues relating to intersectionalities (5/13). Few studies fully reported ethical issues (3/13) or critically examined research relationships (1/13); all that had were led by or had high levels of client involvement. Notably, three studies were conducted by clinical researchers employed at the sites being explored and one by a researcher employed by the service under study.

### 3.3 Findings

The results are clustered around three elements of the assessment process: the journey to the assessment, at the assessment and after the assessment, with a number of themes and subthemes identified at each point—Table 2 provides an overview. Quotes directly from research participants are differentiated in the text from author quotes by the use of italics. Table S5 gives a fuller overview of the findings through further exemplar quotations.

**Table 2:** Overview of people's experiences of being assessed for psychological therapies

Phase of the assessment process	Theme, illustrative quote and summary of content	Subtheme, illustrative quote and summary of content
JOURNEY TO THE ASSESSMENT	*Distress and desperation* “The drugs and psychiatrist were not working – I was desperate” (Morris) Desperation and crisis underpinning help‐seeking; legitimacy of claim to support; gratitude	‐
	*Seeking a new approach* “I was tired of being locked up” (McDonagh) Trying to ease one's distress; failure of other approaches; assessment as a last resort	‐
	*Gatekeeping* “[It is] discouraging when it takes courage to ask for help and you are forced to ask again and again” (Hamilton) Courage to seek therapy; barriers to referral; importance of self‐referral	‐
	*A difficult wait* “When patients are most in need and have the least support” (Hamilton) Anticipating the assessment; therapy as a restricted commodity; waiting; information and support needs	‐
AT THE ASSESSMENT	“I wanted to know that I could share an aspect of myself” (McDonagh) Feeling safe to share parts of yourself; beginnings	*Traumatic, cathartic* “You start talking….it's amazing how much emotion is just bubbling under the surface” (Morris) Distress and catharsis; the importance of collaboration; a catalyst for change
		*Opening up, closing down* “You've got to watch what you say. You definitely don't want to show extreme signs of anger or suicidal thoughts” (Danna)Opening up and holding back; the importance of therapeutic relationships
		*Validation, pathologization* “l did not want my lesbianism looked on as pathology. Did not want to be blamed for violence done to me” (McDonagh) Feeling believed; being pathologized; the role of specialist services
		*Social identity* “Understanding my background in order to understand me” (Morris) The impact of social identity; sharing language, sharing backgrounds
		*Staff impact* “I felt that someone understood and cared” (Fornells‐Ambrojo) Assessor qualities; assessing the assessor; receptionists
		*Assessment techniques* “It is difficult and pointless to quantify how I am feeling” (Fornells‐Ambrojo) Positive, negative and ambivalent experiences of techniques
	“I didn't know what rights I had” (McDonagh) Rights, information and agency	*Information giving and gathering* “I didn't know what would happen at all” (Marshall) Information giving and gathering; exercising agency, information needs; information exchange
		*Authority and agency* “The interviewer kept insisting that I answer the questions even though I clearly said I felt uncomfortable” (Hann) Assessor as powerful agent; collaborative assessments
AFTER THE ASSESSMENT	*Another difficult wait* “The coming weeks could not come fast enough” (Danna) Ready for therapy; the pain of waiting	‐
	*Receiving feedback* “I don't want to be an ignorant patient” (Cape)Prerequisite for therapeutic alliance; mixed experiences of written feedback	‐
	*Making choices* “You get what you are given” (Bryant) Lack of informed choice over therapy or therapist; trusting the assessor; choice and socioeconomics	
	*Assessor‐therapist continuity* “I'm not a particularly open person. So for me to do what I did in the first interview, and to have to do that all over again…. I thought that I had started therapy” (Morris) Lack of advance warning; familiarization and consistency; continuity and socioeconomics	
	*Not going on to therapy* “I just needed an answer as to what to do… I just felt absolutely worthless.” (Bryant)The pain of rejection; dropping out	

The SUAG drew strong parallels between the preliminary results, their personal experiences and the findings of broader survivor‐led studies.^30^ There were occasional points of divergence, most notably around experiences of post‐assessment feedback. During the data workshop, the group again felt that the findings reflected their experiences, identifying areas of importance and implications which have informed the discussion.

## 4 THE JOURNEY TO THE ASSESSMENT

### 4.1 Distress and desperation

The drugs and psychiatrist were not working – I was desperate (Morris)

People's reasons for seeking assessments were predominantly captured in four papers,^31^, ^32^, ^33^, ^34^ often conveying a sense of desperation and crisis. Morris summarized people's reasons as a triangulation between (a) current life events (such as divorce); (b) past events and behaviours (eg, childhood sexual abuse/self‐harm); and (c) current feelings and behaviours (eg, anger/eating problems).^31^ Women often tolerated enormous amounts of distress before seeking help.^31^ Despite this, some, men and women, questioned the legitimacy of their right to support or attention^31^, ^35^, ^36^ and felt grateful to receive a service.^31^, ^37^

### 4.2 Seeking a new approach

I was tired of being locked up (McDonagh)

Many people had tried different approaches to easing their distress, including traditional psychological and talking therapies, mental health services, alternative therapies and self‐help. People using specialist services (for women or people diagnosed with personality disorder) often reported negative contacts with psychiatry which motivated them to seek a new approach.^31^, ^32^, ^33^, ^36^ For some using specialist personality disorder services, the service was considered a “last resort” as all prior attempts to engage with mental health services had been unsuccessful.^32^

### 4.3 Gatekeeping

[It is] discouraging when it takes courage to ask for help and you are forced to ask again and again (Hamilton)

Whilst approaching a therapy service often took courage,^31^, ^35^, ^37^ Hann found that people with poor assessment experiences had often struggled with convoluted referrals.^38^ GPs were important gatekeepers and could be a barrier to referral if they lacked knowledge about local services.^35^, ^37^, ^38^, ^39^ For some in contact with mental health services or with negative experiences of psychiatry, self‐referral was an important way of bypassing medical establishments.^31^

### 4.4 A difficult wait

When patients are most in need and have the least support (Hamilton)

Anticipating the assessment was often highly emotional: people described feeling nervous; daunted; scared; relieved; desperate; frightened of rejection; concerned about the legitimacy of their claim to support; hopeless, “it's this or nothing”; hopeful of being “cured”; and eager to begin.^31^, ^32^, ^34^, ^38^

There was a sense that therapy is “a restricted commodity”,^31^ particularly in the context of UK funding cuts.^38^ Consequently, people appreciated short waits between referral and assessment.^31^, ^35^, ^37^, ^38^ For others, having their hopes raised and then waiting for an assessment—sometimes extensively—at a time of intense distress were very difficult^32^, ^37^, ^38^, ^39^ and caused some to drop out of the process. There was a need for basic contacts (for instance, a letter confirming a waiting list place) and support, although not everyone wanted interim support.^32^, ^35^, ^37^, ^38^, ^39^ The length of the wait was easier to bear if people had been experiencing difficulties for a long time, or were waiting for a therapist of their choice (eg, a black female therapist).^31^

## 5 AT THE ASSESSMENT

### 5.1 I wanted to know that I could share an aspect of myself (McDonagh)

Fundamental to assessments is that the client shares something of themselves with the assessor, often relating to difficult and painful experiences. This requires support, a sense of trust and safety, and, at times, a shared identity, and can cause additional trauma or spark the beginnings of catharsis.

#### 5.1.1 Traumatic, cathartic

You start talking….it's amazing how much emotion is just bubbling under the surface (Morris)

Undergoing an assessment was often an emotional experience. Whilst this could be cathartic and validating, sparking new insights,^31^, ^34^, ^36^ it could also be painful and traumatic, raising difficult and painful issues without sufficient support.^32^ A significant determining factor appeared to be the degree of collaboration: explicitly collaborative assessments conducted across multiple sessions were often experienced as empowering,^34^, ^36^ whilst assessments with multiple sessions that were *done to* a person were more often experienced as traumatic^32^; this was in the context of the latter often being a “last resort”.^32^ Positive assessments could inspire hope and be a catalyst for change,^31^, ^34^, ^36^ providing a foundation and direction for therapy.^34^ Good outcomes were sometimes traceable to assessments.^31^

#### 5.1.2 Opening up, closing down

You've got to watch what you say. You definitely don't want to show extreme signs of anger or suicidal thoughts. (Danna)

Danna described participants’, “ambivalence … between feeling compelled to share difficult aspects of themselves … but simultaneously heeding the natural tendency to protect oneself due to the implicit vulnerability that opening oneself up to others entails”.^34^ A participant who had previously been hospitalized following an assessment was “definitely guarded”.^34^ Similarly, Morris found that whilst some women opened up, for others it was important to establish a therapeutic relationship first; she describes one woman disclosing experiences of childhood sexual abuse in the assessment but waiting a year to disclose bulimia because this was experienced as so shameful.^31^

#### 5.1.3 Validation, pathologization

l did not want my lesbianism looked on as pathology. Did not want to be blamed for violence done to me (McDonagh)

Feeling believed and heard had a validating impact,^31^, ^36^, ^38^ whilst feeling unheard was at times experienced as a betrayal.^39^ For some using women's and “personality disorder” services, feeling validated or pathologized was entwined with previous experiences of psychiatry.^31^, ^32^, ^33^, ^36^

McDonagh found that most “traditional [non‐feminist] therapists denied the women's histories of sexual abuse, failed to ask about sexual abuse … failed to incorporate such information into assessments when it was provided… [and] tended to pathologise the effects of the sexual abuse”.^33^ Consequently, some women sought women's/feminist therapy that would “see beyond psychiatric diagnoses to the person and their life experiences underneath”.^31^ When women felt that therapy services were becoming medical, they could lose trust; for instance, one woman who was asked for her psychiatrist's details felt pathologized and disengaged shortly after starting therapy.^31^ Similarly, Bryant found that some people selected a service because it could respond to a particular issue (eg, sexual abuse, addiction) or because female counsellors were available (preferred by a minority of both women and men).^35^

In their analysis, Lavie‐Ajayi found that the client's (Sima's) complex ecological accounts of her difficulties were ignored in favour of the therapist's (Rivka's) individualist interpretation.^40^ Sima “tried to balance the expectation to be a ‘good client’ and not challenge the medical internal discourse while retaining her right not to accept the treatment recommendations”. Post‐assessment she asserted:

Instead of insisting and … get locked on psychiatric medication, one could think beyond that … if she had helped me to solve the problem at my workplace, she would have cured me.

#### 5.1.4 Social identity

Understanding my background in order to understand me (Morris)

Through their analysis, Lavie‐Ajayi concluded that gender, ethnicity and class had affected the assessment encounter between Sima and Rivka.^40^ Morris also found that social identity shaped assessments: being assessed by someone with a shared cultural background and/or language meant women could be understood in their own words and contexts.^31^

#### 5.1.5 Staff impact

I felt that someone understood and cared (Fornells‐Ambrojo)

Morris found that therapists were typically described in two ways, “those who appeared friendly, welcoming and encouraging and those who were perceived as … cold, neutral or very quiet”.^31^ People who found the assessment difficult typically had a neutral or quiet therapist. Where this occurred, the person sometimes went on to dislike their therapist or group, often disengaging after a few sessions. Conversely, experiencing kindness could be “heart‐warming”,^36^ creating a sense of hope,^37^ with the manner of giving feedback either opening or closing opportunities for self‐reflection.^34^ Staff could also be experienced as patronizing, or failing to respond to people as individual's, preventing engagement.^39^ McDonagh found that women who sought feminist therapy were more likely to interview their prospective therapists, effectively assessing their assessor.^33^

Receptionists also impacted on people's experiences, Hamilton concluding,

Reception staff are vital to creating a positive atmosphere in the service. We had reports of friendly, helpful and efficient staff, but we also heard about staff who were snappy and unfriendly. People talked about the courage involved in coming to the IAPT service. What might seem like a small gesture on the part of individual staff members may have a huge impact on whether patients feel relaxed in attending the service. 37

#### 5.1.6 Assessment techniques

It is difficult and pointless to quantify how I am feeling (Fornells‐Ambrojo)

Techniques—ranging from Rorshach to symptom measures—were often experienced positively, helping people express themselves (particularly where spoken communication was difficult); gain clarity around feelings; positively reframe issues; and open space for thinking and reflection.^34^, ^36^, ^41^ Where people were ambivalent or disliked techniques, it was typically because they were lengthy, challenging, upsetting, inflexible or difficult to engage with.^31^, ^32^, ^33^, ^39^, ^40^ Some were sceptical about computer judgements and preferred to talk, “I mean, it's like, I could have told you that”.^34^ Whilst participants in De Seager were positive about testing, one of the only negative comments across the study was a person wondering “why all these tests”.^36^

### 5.2 I didn't know what rights I had (McDonagh)

There is an inherent, typically unacknowledged power imbalance between clients and assessors. Whilst there are individual differences in the extent to which people wish to exercise agency and choice, people nonetheless have basic rights and information needs that are not always met.

#### 5.2.1 Authority and agency

The interviewer kept insisting that I answer the questions even though I clearly said I felt uncomfortable (Hann)

Assessors were often experienced as the people with “authority” who “know best”.^31^ Whilst this could be positive, resulting in direction from an expert about whether and which therapy to try,^31^, ^37^ for others it compromised agency. For instance, Sima (Lavie‐Ajayi) had to navigate a difficult path through her assessment without overtly challenging Rivka's discourse.^40^ Conversely, explicitly collaborative assessments were typically valued, engendering a sense of equality or involvement which enhanced agency.^34^, ^36^

#### 5.2.2 Information giving and gathering

I didn't know what would happen at all (Marshall)

The provision of information can be one‐way, or “more akin to a dialogue than to unilateral information gathering”.^36^ The extent to which clients exercised agency varied: whilst some were happy to be guided by the therapist,^31^, ^37^ others asked questions,^31^, ^33^ or wanted to but felt unable.^31^ Information needs included: alternative local therapy services; practicalities; and details of the therapeutic process.^35^ Lacking information created uncertainty^39^ and could reinforce a sense of secrecy surrounding therapy^35^ and feel damaging.^31^ In particular, not being informed about the “rules of therapy” at the assessment (such as the use of silences) could cause people to subsequently disengage.^35^ Further, it could mean that boundary breaches were not recognized, compromising rights:

I wish I had known more because my first therapist over‐stepped my boundaries and I didn't know what rights I had. 33

Conversely, sharing good, clear information was valued,^33^, ^35^, ^37^ resulting in people feeling better prepared and able to exercise choice.^35^

## 6 AFTER THE ASSESSMENT

### 6.1 Another difficult wait?

The coming weeks could not come fast enough (Danna)

Whilst some people left the assessment hopeful and “Willing to go through the fire”*,* 34 often with the understanding that therapy would be a difficult process, others felt “opened up” and had a potentially difficult wait for therapy.^36^

### 6.2 Receiving feedback

I don't want to be an ignorant patient (Cape)

Some studies described the impact of receiving face‐to‐face or written feedback about the assessment and its outcomes. Face‐to‐face feedback created opportunities to challenge misinterpretations and for many was a prerequisite for a trusting relationship.^34^, ^42^ Whilst written feedback could mean people felt listened to, understood and validated,^36^, ^42^ or made their problems feel contained and manageable, for others it was intensely distressing.^42^

### 6.3 Making choices

You get what you are given (Bryant)

There were few opportunities to discuss therapy options^35^ and little choice about which therapy or therapist people received post‐assessment.^31^, ^35^, ^37^, ^39^ Some accepted this, trusting their referrer or assessor, or feeling grateful to receive any service, whilst others felt disappointed and unable to make informed choices.^31^, ^35^, ^37^ Barber reported that one person felt that being assigned to the wrong service post‐assessment was a “waste of money, time, resources … putting people to the right service is fundamental”.^43^ People with means were able to exercise choice, selecting their therapist privately.^33^

### 6.4 Assessor ‐therapist continuity

I'm not a particularly open person. So for me to do what I did in the first interview, and to have to do that all over again…. I thought that I had started therapy (Morris)

Many were unaware that their assessor would not be their therapist, and this could be upsetting. People who had the same assessor and therapist valued the familiarization and consistency.^34^, ^37^ This issue was avoided where people had the means to purchase therapy.^33^

### 6.5 Not going on to therapy

I just needed an answer as to what to do… I just felt absolutely worthless. (Bryant)

Bryant explored the experiences of four people who were “willing to go through the fire” but were not offered therapy.^35^ All had long‐term mental health service contact histories. None understood why they were declined therapy and none appeared to have received information about alternative services. People consequently felt powerless; angry; frustrated; rejected; bewildered; disappointed; hopeless; and worthless.

Marshall explored the experiences of 14 people who disengaged from IAPT, finding that lengthy waits without support, rigid assessments, a lack of information about therapy, patronizing communication styles and a lack of individualized approaches were all contributory factors.^39^

Whilst many who had taken the decision to have an assessment had therefore decided to accept therapy once offered,^31^ others decided not to proceed with therapy. Reasons for declining included because people had needed help urgently; were no longer well enough to engage in therapy; or had found alternative support.^35^, ^38^

## 7 DISCUSSION

This review synthesizes qualitative research on people's experiences of being assessed for psychological therapies; the findings can usefully inform best practice around assessments and can also be understood through trauma‐informed (TIA) principles.

In fully understanding people's experiences, a process‐based conceptualization was adopted, rather than seeing assessments as one‐off encounters. This process arguably begins when people are finding the courage to seek an assessment and ends when people are waiting—typically without support—for therapy to begin, or attempting to recover from a rejection. This mirrors the conceptualization adopted in the TIA literature and proposed elsewhere^44^, ^45^ and enables a greater understanding of the ways in which assessments impact people's experiences.

The extent of collaboration, along with therapeutic alliance,^46^ can determine whether clients have positive assessment experiences that are empowering, facilitate change and promote agency and hope, or negative experiences that incite distress, powerlessness and hopelessness. Collaboration is also a fundamental principle of TIAs, meaning that the inherent power imbalance between staff and clients is understood, with relationships based on mutuality, respect, trust, connection and hope.^12^ Trauma‐informed assessments are shared, collaborative processes which seek to discuss and clarify connections, sequences, coping adaptations and strengths.^14^ It is possible that such assessments could reduce dropout and improve experience through creating a high‐quality initial encounter, with further research warranted. Whilst the need for collaboration may seem self‐evident, explicitly collaborative assessments were not the norm. Moreover, across psychiatric services, service users typically feel that they are done *to*, rather than *with*.^47^

Whilst collaborative assessments may be an important example of good practice, the collaborative assessment studies we reviewed were conducted by clinicians employed in those services.^28^ Consequently, independent service user research is needed as it is uniquely positioned to understand client experience.^48^ There is also a danger that therapist‐led research could interpret client's experiences through a therapeutic, rather than research lens; this could, for instance, result in experiences being dismissed as transference, or for what they reveal about a person's psyche, rather than what they reveal about a service.^17^ Referring to client involvement, Trivedi writes,

getting service users to identify for themselves the reasons why they might have dropped out and then working with them to address the issues could help make services more ‘user‐friendly’ with a subsequent fall in dropout rates. 17

Seeking, waiting for, undergoing and moving on from an assessment can be intensely distressing, and the desperation underpinning help‐seeking, as well as the potentially negative impacts of assessments, should not be underestimated. Within this, the tension between “opening up,” often to persuade an assessor that you deserve therapy, and being “guarded,” for instance to protect oneself from overwhelm, can be understood as rational struggles. In line with TIAs, assessors must carefully manage disclosures and attend to emotional safety.^15^ Waiting for an assessment, or for therapy to begin, was particularly difficult for people in intense distress and could cause people to disengage. Services should consider the possibilities for rapid assessment and interim support, as well as support where therapy is not offered (potentially as simple as onward referrals).

Our review also points to the vital role of women's therapy services, particularly for women who have experienced gender‐based violence and/or who self‐identify as lesbian. In a climate of funding uncertainty, particularly in the UK, this finding is notable.^49^ We also found that where people had experienced coercive psychiatry, assessments that felt medicalizing prevented engagement. As a minimum, assessors should explain why they are requesting information, consistent with TIAs.^14^ Beyond this, whilst there clearly needs to be a fit between the frameworks of understanding held by the client, the assessor and the subsequent therapist, in practice this fit is often restricted to those with socioeconomic means, with UK NHS clients sometimes feeling “you get what you're given.”

Our review also highlighted the link between information and rights, with people having clear information needs at each point of the assessment process. Yet there was a sense that traditional psychotherapies in particular can be secretive, with unspoken “rules”.^17^ Disclosing these rules within the assessment process enables informed choice, can prevent disengagement, and empowers people to understand rule breaches, particularly pertinent in the light of the #MeToo movement (a social media campaign raising awareness about the prevalence of sexual violence and harassment^50^). Ideally, people undergoing assessment would be informed of an independent person or organization that they could contact to discuss concerns.^51^

Whilst there is an inherent difficulty in building therapeutic alliance within one‐off encounters, we nonetheless found that people were less likely to disengage if their assessor demonstrated warmth, kindness and collaboration. Receptionists also impacted on people's experiences. Within TIAs, it is understood that all staff, including clinical, domestic and administrative, shape people's experiences and consequently all staff receive TIA training.^12^

### 7.1 Study limitations and further research

Methodological limitations include that some review work, including the thematic synthesis, was conducted by one reviewer. However, the review had high levels of service user involvement throughout which can enhance quality.^52^ The emerging synthesis was discussed with the SUAG using reflexive techniques to understand the interplay between our experiences and data interpretations. As thematic synthesis is inherently subjective,^53^ others may have arrived at different analytical accounts.

Quality appraisal found that intersectionalities, ethics and research relationships were explored infrequently. Consequently, we are unable to report the experiences of people from minority communities. Future research should employ a critical understanding of research relationships, including the experiences of diverse populations. Given the unique importance of women's services, future research should also consider experiences within services accessed by social identity (eg, for people who identify as LGBTQ) or experience (eg, sexual violence survivors).

The majority of studies (8/13) had not been peer reviewed. This may be because collaborative and service user‐led research has not historically entered mainstream journals, only recently gaining recognition as a valid form of enquiry.^54^ Interestingly, non‐peer‐reviewed literature typically scored higher in the quality assessment.

The majority of papers were from the UK which has the NHS and IAPT programme. This has shaped our findings, for instance around socioeconomic access to services. Future reviews should include literature beyond English language, search a broader range of databases and conduct wider literature calls.

Descriptions of the assessment process are variable, with the majority of studies not reporting the specialty or approach of the assessor/service, the assessment form, its duration, and trauma enquiries and disclosures. This makes it difficult to connect client experience to therapy modalities and the technical question of how assessments are conducted, limiting the inferences that can be drawn and highlighting a need for further research.

Finally, we did not explore assessors’ experiences. Further research investigating assessments as a dyadic interplay between two actors would enable a fuller account of assessment processes.

## 8 CONCLUSIONS

This review aimed to understand adults’ experiences of undergoing psychological assessment. The findings were understood within TIAs, including those relating to the emotional impact of assessments, information and support needs, rights, pathologization, socioeconomic restrictions, intersectionalities and collaboration. Whilst the need for collaboration may appear self‐evident, explicitly collaborative assessments were not the norm and independent service user research is needed. Given the focus of this review, our gaps in understanding and the quality of papers, future research directions have been suggested, emphasizing the importance of understanding the assessment from dyadic and multiple perspectives, including that of minority groups.

## CONFLICT OF INTEREST

The authors declare that they have no conflicts of interest.

## AUTHOR CONTRIBUTIONS

AS, SCl, SCa, JC, SG involved in protocol development. EJ, SCl, KG, AS involved in data searching and screening. AS involved in data extraction and quality appraisal. AS, SCl and SCa involved in data synthesis. AS, SCl, KG, EJ, SC, JC and SG involved in writing.

## References

[1] NHS Digital . Adult Psychiatric Morbidity Survey: Survey of mental health and wellbeing, England, 2014 29th September 2016. https://digital.nhs.uk/catalogue/PUB21748. Accessed April 3rd, 2018.

[2] NHS Digital . Psychological Therapies: Annual report on the use of IAPT services – England, 2016‐7. 30th November 2017. http://www.digital.nhs.uk/catalogue/PUB30157. Accessed April 3rd, 2018.

[3] NHS England . Implementing the Five Year Forward View for Mental Health. London; 2016 https://www.england.nhs.uk/wp-content/uploads/2016/07/fyfv-mh.pdf. Accessed April 3rd, 2018

[4] Teglasi H . Essentials of TAT and Other Storytelling Assessments. Series: Essentials of Psychological Assessment. Hoboken, NJ: Wiley; 2010.

[5] Kroenke K , Spitzer R , Williams J . The PHQ‐9: validity of a brief depression severity measure. J Gen Intern Med. 2001;16:606‐613. 11556941 10.1046/j.1525-1497.2001.016009606.x PMC1495268

[6] Finn SE , Tonsager ME . Information‐gathering and therapeutic models of assessment: complementary paradigms. Psychol Assess. 1997;9:374‐385. 10.1037/1040-3590.9.4.374.

[7] Milner J , O'Byrne P . How do counsellors make client assessments? Couns Psychother Res. 2003;3:139‐145.

[8] Nakash O , Alegria M . Examination of the role of implicit clinical judgments during the mental health intake. Qual Health Res. 2012;23:645‐654. 10.1177/1049732312471732 PMC6365967 23282797

[9] Berk M , Parker G . The elephant on the couch: side‐effects of psychotherapy. Aust N Z J Psychiatry. 2009;43:787‐794. 19670051 10.1080/00048670903107559

[10] Crawford M , Thana L , Farquharson L , et al. Patient experience of negative effects of psychological treatment: results of a national survey. Br J Psychiatry. 2016;208:260‐265. 26932486 10.1192/bjp.bp.114.162628

[11] Hardy GE , Bishop‐Edwards L , Chambers E , et al. Risk factors for negative experiences during psychotherapy. Psychother Res. 2017 https://www.tandfonline.com/doi/abs/10.1080/10503307.2017.1393575?journalCode=tpsr20 10.1080/10503307.2017.1393575 29078740

[12] Sweeney A , Clement S , Filson B , Kennedy A . Trauma‐informed mental healthcare in the UK: what is it and how can we further its development? Ment Health Rev J. 2016;21:174‐192.

[13] Substance Abuse and Mental Health Services Administration . Trauma‐Informed Care in Behavioral Health Services. Treatment Improvement Protocol (TIP) Series 57. HHS Publication No. (SMA) 13‐4801. Rockville, MD: Substance Abuse and Mental Health Services Administration; 2014. 24901203

[14] Harris M , Fallot R . Using Trauma Theory to Design Service Systems. New Directions for Mental Health Services. San Francisco, CA: Jossey‐Bass; 2001.

[15] Ferentz L . Trauma‐Informed Assessments. Parts 1‐8. Psychology Today; October 2015. https://www.psychologytoday.com/blog/healing-trauma-s-wounds/201510/trauma-informed-assessments-part-1. Accessed April 3rd, 2018.

[16] Elliot D , Bjelajac P , Fallot R , Markoff L , Glover Reed B . Trauma‐informed or trauma‐denied: principles and implementation of trauma‐informed services for women. J Community Psychol. 2005;33:461‐477.

[17] Trivedi P . Service user involvement, ethics and power in therapy services In: Tribe R, Morrissey J, eds. The Handbook of Professional, Research and Ethical Practice for Psychologists, Counsellors and Psychotherapists, 3rd edn Hoboken, NJ: Wiley‐Blackwell (forthcoming).

[18] Godin K , Stapleton J , Kirkpatrick S , Hanning R , Leatherdale T . Applying systematic review search methods to the grey literature: a case study examining guidelines for school‐based breakfast programs in Canada. Syst Rev. 2015;4:138. 26494010 10.1186/s13643-015-0125-0 PMC4619264

[19] Clark JP . How to peer review a manuscript In: Godlee F, Jefferson T, eds. Peer review in Health Sciences, 2nd edn London, UK: BMJ Books; 2003:219‐235.

[20] Critical Appraisal Skills Programme . CASP Qualitative Research Checklist. [online]. 2017 http://docs.wixstatic.com/ugd/dded87_25658615020e427da194a325e7773d42.pdf. Accessed April 3rd, 2018.

[21] Rees RW , Caird J , Dickson K , Vigurs C , Thomas J . ‘It's on your conscience all the time’: a systematic review of qualitative studies examining views on obesity among young people aged 12–18 years in the UK. BMJ Open. 2014;4:e004404. 10.1136/bmjopen-2013-004404 PMC4010837 24785398

[22] Brunton G , Wiggins M , Oakley A . Becoming a Mother: A Research Synthesis of Women's Views on the Experience of First‐time Motherhood. London, UK: EPPI Centre, Social Science Research Unit, Institute of Education, University of London; 2011.

[23] Harden A , Brunton G , Fletcher A , Oakley A , Burchett H , Backhans M . Young People, Pregnancy and Social Exclusion: A Systematic Synthesis of Research Evidence to Identify Effective, Appropriate and Promising Approaches for Prevention and Support. London, UK: EPPI‐Centre, Social Science Research Unit, Institute of Education, University of London; 2006.

[24] Thomas J , Sutcliffe K , Harden A , et al. Children and Healthy Eating: A Systematic Review of Barriers and Facilitators. London, UK: EPPI‐Centre, Social Science Research Unit, Institute of Education, University of London; 2003.

[25] Malpass A , Shaw A , Sharp D , et al. “Medication career” or “Moral career”? The two sides of managing antidepressants: a meta‐ethnography of patients’ experience of antidepressants. Soc Sci Med. 2009;68:154‐168. 19013702 10.1016/j.socscimed.2008.09.068

[26] Feder GS , Hutson M , Ramsay J , Taket AR . Women exposed to intimate partner violence: expectations and experiences when they encounter health care professionals: a meta‐analysis of qualitative studies. Arch Intern Med. 2006;9(166):22‐37. 10.1001/archinte.166.1.22 16401807

[27] Thomas J , Harden A . Thematic Synthesis. Southampton, UK: ESRC National Centre for Research Methods; 2008.

[28] Shimmin C , Wittmeier KDM , Lavoie JG , Wicklund ED , Sibley KM . Moving towards a more inclusive patient and public involvement in health research paradigm: the incorporation of a trauma‐informed intersectional analysis. BMC Health Serv Res. 2017;17:539. 28784138 10.1186/s12913-017-2463-1 PMC5547533

[29] Scope A , Uttley L , Sutton A . A qualitative systematic review of service user and service provider perspectives on the acceptability, relative benefits, and potential harms of art therapy for people with non‐psychotic mental health disorders. Psychol Psychother. 2017;90:25‐43. 27257043 10.1111/papt.12093

[30] Carr S , Holley J , Hafford‐Letchfield T , et al. Mental health service user experiences of targeted violence and hostility and help‐seeking in the UK: a scoping review. Glob Ment Health. 2017;4:e25. 10.1017/gmh.2017.22 PMC5733370 29270301

[31] Morris B . Discovering Bits and Pieces of me: Research Exploring Women's Experiences of Psychoanalytical Psychotherapy. London, UK: Women's Therapy Centre; 2005.

[32] Crawford M , Rutter D , Price K , et al. Learning the lessons: A multimethod evaluation of dedicated community‐based services for people with personality disorder. Report for the National Co‐ordinating Centre for NHS Service Delivery and Organisation R&D (NCCSDO). London, UK: Queen's Printer and Controller of HMSO; 2007.

[33] McDonagh D . Exploring Client Perspectives of Therapy: Women survivors in feminist therapy. A thesis submitted in conformity with the requirements for the Degree Doctor of Philosophy. University of Toronto, Canada: Department of Adult Education, Community Development and Counselling Psychology, Ontario Institute for Studies in Education; 1997.

[34] Danna J . Therapist and client experience of collaborative psychological assessment: A qualitative study. A Dissertation Submitted to the McAnulty College and Graduate School of Liberal Arts. Pittsburgh: Duquesne University; 2011.

[35] Bryant L , Beckett J , Clarke J , Shilling L . Pathways to therapy: service‐user views of accessing psychological therapies in Leeds. Report for the Leeds Primary Care Trust Modernisation Team. University of Leeds, UK: Academic Unit of Psychiatry and Behavioural Sciences, Institute of Health Sciences; 2007.

[36] De Saeger H , Bartak A , Eder E‐E , Kamphuis JH . Memorable experiences in therapeutic assessment: inviting the patient's perspective following a pretreatment randomized controlled trial. J Pers Assess. 2016;98:472‐479. 26829376 10.1080/00223891.2015.1136314

[37] Hamilton S , Hicks A , Sayers R , et al. A user‐focused evaluation of IAPT services in London. Report for Commissioning Support for London. London, UK: Rethink; 2011.

[38] Hann A , Hemming L , Billsborough J , et al. A Service User Evaluation of the IAPT for SMI Demonstration Sites. London, UK: McPin Foundation and NHS England; 2015.

[39] Marshall D , Quinn C , Child S , et al. What IAPT services can learn from those who do not attend. J Ment Health. 2016;25:410‐415. 26635236 10.3109/09638237.2015.1101057

[40] Lavie‐Ajayi M , Nakash O . “If she had helped me to solve the problem at my workplace, she would have cured me”: a critical discourse analysis of a mental health intake. Qual Soc Work. 2017;16:60‐77.

[41] Fornells‐Ambrojo M , Johns L , Onwumere J , et al. Experiences of outcome monitoring in service users with psychosis: findings from an Improving Access to Psychological Therapies for people with Severe Mental Illness (IAPT‐SMI) demonstration site. Br J Clin Psychol. 2017;56:253‐272. 28493592 10.1111/bjc.12136

[42] Cape J , Harvey K , Johnson S , Linke S . Patients’ views of the letters their psychiatrists and psychologists send to referrers. J Ment Health. 2005;14:369‐382.

[43] Barber N , Bartnik J , Collins F , et al. Evaluating Sutton Uplift. A Service User Led Evaluation. London, UK: St Georges, University of London; 2017.

[44] Waddell M . Assessing adolescents: finding space to think In: Rustin M, Quagliata E, eds. Assessment in Child Psychotherapy. London, UK: Karnac; 2000:145‐161.

[45] Hird M . Service user involvement in mental health assessment: comparing people's experiences of mental health triage assessments with theoretical perspectives on user involvement. Int J Psychiatr Nurs Res. 2007;13:1561‐1577. 17927024

[46] Catty J . ‘The vehicle of success’: theoretical and empirical perspectives on the therapeutic alliance in psychotherapy and psychiatry. Psychol Psychother. 2004;77:255‐272. 15193196 10.1348/147608304323112528

[47] Castillo H , Ramon S . “Work with me”: service users’ perspectives on shared decision making in mental health. Ment Health Rev J. 2017;22:166‐178. 10.1108/MHRJ-01-2017-0005.

[48] Beresford P . It's Our Lives: A Short Theory of Knowledge, Distance and Experience. London, UK: OSP for Citizens Press in Association with Shaping Our Lives; 2003.

[49] Ferraro D . Psychology in the age of austerity. Psychother Polit Int. 2016;14:17‐24.

[50] Key K . If Your Therapist Harasses You: #MeToo. Psychology Today, October 2017 https://www.psychologytoday.com/blog/counseling-keys/201710/if-your-therapist-harasses-you-metoo. Accessed April 3rd, 2018.

[51] Faulkner A . On power and privacy. Member blog, National Survivor User Network; 2017 https://www.nsun.org.uk/on-power-and-privacy. Accessed April 3rd, 2018.

[52] Smith E , Donovan S , Beresford P , et al. Getting ready for user involvement in a systematic review. Health Expect. 2009;12:197‐208. 19236632 10.1111/j.1369-7625.2009.00535.x PMC5060487

[53] Bucci S , Roberts NH , Danquah AN , Berry K . Using attachment theory to inform the design and delivery of mental health services: a systematic review of the literature. Psychol Psychother. 2015;88:1‐20. 24729543 10.1111/papt.12029

[54] Rose D , Carr S , Beresford P . ‘Widening cross‐disciplinary research for mental health’: what is missing from the Research Councils UK mental health agenda? Disabil Soc. 2018;33:476‐481.

