# Supplementary material for: A systematic review of qualitative studies of adults’ experiences of being assessed for psychological therapies
Source: Health Expect. 2019 Jan 8;22(2):133–48. doi: 10.1111/hex.12844 (PMC6433316; doi:10.1111/hex.12844)
Supplement: Supplementary file 1 [file HEX-22-133-s001.docx]

# Data supplements

*Please note that the tables within this document are intended as supporting information for online publication only.*

| **Table S1: Final search terms for peer reviewed electronic databases** | | | |
| --- | --- | --- | --- |
| ***Experiential evidence*** | ***Method*** | ***Phenomena*** | ***Area of health*** |
| Service user experience  ----------  Boolean operator ‘or’ used within grouped terms | Qualitative  ----------  Boolean operator ‘or’ used within grouped terms | Assessment  ----------  Boolean operator ‘or’ used within grouped terms | Mental health  ----------  Boolean operator ‘or’ used within grouped terms |
| Boolean operator ‘and’ used between grouped terms | | | |
| Service user; expert by experience; patient experience; consumer experience; client experience; service user satisfaction; consumer satisfaction; client satisfaction; service user perception; patient perception; consumer perception; client perception; service user beliefs; patient beliefs; consumer beliefs; client beliefs; service user perspectives; patient perspectives; consumer perspectives; client perspectives; service user attitudes; patient attitudes; consumer attitudes; client attitudes; service user opinions; patient opinions; consumer opinions; client opinions; service user narrative; patient narrative; consumer narrative; client narrative. | Interviews; focus groups; ethnography; phenomenology; hermeneutic; grounded theory; content analysis; thematic analysis; discourse analysis; conversation analysis; framework analysis; narrative analysis; lived experience. | Assessment; assessed; initial consultation; new consultation; first consultation. | Mental health; mental health services; mental disorders; mentally ill; mental illness; psychiatry; psychology; well-being; depression; schizophrenia; bipolar; anxiety; personality disorder; phobia; panic; emotional; self-harm; self-injury; talking therapy; increasing access to psychological therapies; IAPT; cognitive behavioural therapy; cognitive behavioral therapy; CBT; dialectical behaviour therapy; dialectical behavior therapy; DBT; counselling; psychotherapy; psychoanalysis; psychodynamic; interpersonal therapy; group therapy. |

| **Table S2: Example of a search strategy in PsychINFO (1967 to July Week 5 2017) (Ovid)** |
| --- |
| 1 Consumer Satisfaction/ (4358)  2 Consumer Attitudes/ (12860)  3 patient experience*.mp. [mp=title, abstract, heading word, table of contents, key concepts, original title, tests & measures] (2267)  4 consumer experience*.mp. [mp=title, abstract, heading word, table of contents, key concepts, original title, tests & measures] (282)  5 client experience*.mp. [mp=title, abstract, heading word, table of contents, key concepts, original title, tests & measures] (376)  6 expert by experience.mp. [mp=title, abstract, heading word, table of contents, key concepts, original title, tests & measures] (15)  7 service user satisfaction.mp. [mp=title, abstract, heading word, table of contents, key concepts, original title, tests & measures] (30)  8 Client Satisfaction/ (4919)  9 Client Attitudes/ (15094)  10 patient satisfaction.mp. [mp=title, abstract, heading word, table of contents, key concepts, original title, tests & measures] (4274)  11 consumer satisfaction.mp. [mp=title, abstract, heading word, table of contents, key concepts, original title, tests & measures] (5152)  12 client satisfaction.mp. [mp=title, abstract, heading word, table of contents, key concepts, original title, tests & measures] (6291)  13 service user perception*.mp. [mp=title, abstract, heading word, table of contents, key concepts, original title, tests & measures] (17)  14 patient perception*.mp. [mp=title, abstract, heading word, table of contents, key concepts, original title, tests & measures] (1305)  15 consumer perception*.mp. [mp=title, abstract, heading word, table of contents, key concepts, original title, tests & measures] (1065)  16 client perception*.mp. [mp=title, abstract, heading word, table of contents, key concepts, original title, tests & measures] (557)  17 service user belief*.mp. [mp=title, abstract, heading word, table of contents, key concepts, original title, tests & measures] (2)  18 patient belief*.mp. [mp=title, abstract, heading word, table of contents, key concepts, original title, tests & measures] (218)  19 consumer belief*.mp. [mp=title, abstract, heading word, table of contents, key concepts, original title, tests & measures] (100)  20 client belief*.mp. [mp=title, abstract, heading word, table of contents, key concepts, original title, tests & measures] (55)  21 service user perspective*.mp. [mp=title, abstract, heading word, table of contents, key concepts, original title, tests & measures] (112)  22 patient perspective*.mp. [mp=title, abstract, heading word, table of contents, key concepts, original title, tests & measures] (1019)  23 consumer perspective*.mp. [mp=title, abstract, heading word, table of contents, key concepts, original title, tests & measures] (409)  24 client perspective*.mp. [mp=title, abstract, heading word, table of contents, key concepts, original title, tests & measures] (244)  25 service user attitude*.mp. [mp=title, abstract, heading word, table of contents, key concepts, original title, tests & measures] (6)  26 patient attitude*.mp. [mp=title, abstract, heading word, table of contents, key concepts, original title, tests & measures] (1094)  27 consumer attitude*.mp. [mp=title, abstract, heading word, table of contents, key concepts, original title, tests & measures] (13126)  28 client attitude*.mp. [mp=title, abstract, heading word, table of contents, key concepts, original title, tests & measures] (15218)  29 service user opinion*.mp. [mp=title, abstract, heading word, table of contents, key concepts, original title, tests & measures] (5)  30 patient opinion*.mp. [mp=title, abstract, heading word, table of contents, key concepts, original title, tests & measures] (86)  31 consumer opinion*.mp. [mp=title, abstract, heading word, table of contents, key concepts, original title, tests & measures] (89)  32 client opinion*.mp. [mp=title, abstract, heading word, table of contents, key concepts, original title, tests & measures] (21)  33 service user narrative*.mp. [mp=title, abstract, heading word, table of contents, key concepts, original title, tests & measures] (7)  34 patient narrative*.mp. [mp=title, abstract, heading word, table of contents, key concepts, original title, tests & measures] (175)  35 consumer narrative*.mp. [mp=title, abstract, heading word, table of contents, key concepts, original title, tests & measures] (32)  36 client narrative*.mp. [mp=title, abstract, heading word, table of contents, key concepts, original title, tests & measures] (68)  37 or/1-36 (44347)  38 Qualitative Research/ (7477)  39 qualitative.mp. [mp=title, abstract, heading word, table of contents, key concepts, original title, tests & measures] (129376)  40 exp INTERVIEWS/ (11718)  41 NARRATIVES/ (17029)  42 PHENOMENOLOGY/ (11841)  43 Grounded Theory/ (3244)  44 HERMENEUTICS/ (1900)  45 ETHNOGRAPHY/ (7584)  46 interview*.mp. [mp=title, abstract, heading word, table of contents, key concepts, original title, tests & measures] (331365)  47 focus group*.mp. [mp=title, abstract, heading word, table of contents, key concepts, original title, tests & measures] (28371)  48 ethnograph*.mp. [mp=title, abstract, heading word, table of contents, key concepts, original title, tests & measures] (24498)  49 phenomenolog*.mp. [mp=title, abstract, heading word, table of contents, key concepts, original title, tests & measures] (35433)  50 hermeneutic*.mp. [mp=title, abstract, heading word, table of contents, key concepts, original title, tests & measures] (6349)  51 grounded theory.mp. [mp=title, abstract, heading word, table of contents, key concepts, original title, tests & measures] (13033)  52 content analy*.mp. [mp=title, abstract, heading word, table of contents, key concepts, original title, tests & measures] (23685)  53 exp Content Analysis/ (12344)  54 thematic analy*.mp. [mp=title, abstract, heading word, table of contents, key concepts, original title, tests & measures] (7979)  55 discourse analy*.mp. [mp=title, abstract, heading word, table of contents, key concepts, original title, tests & measures] (11035)  56 conversation analy*.mp. [mp=title, abstract, heading word, table of contents, key concepts, original title, tests & measures] (2088)  57 framework analy*.mp. [mp=title, abstract, heading word, table of contents, key concepts, original title, tests & measures] (538)  58 framework analy*.mp. [mp=title, abstract, heading word, table of contents, key concepts, original title, tests & measures] (538)  59 lived experience*.mp. [mp=title, abstract, heading word, table of contents, key concepts, original title, tests & measures] (10153)  60 38 or 39 or 40 or 41 or 42 or 43 or 44 or 45 or 46 or 47 or 48 or 49 or 50 or 51 or 52 or 53 or 54 or 55 or 56 or 57 or 58 or 59 (481267)  61 assessment*.mp. [mp=title, abstract, heading word, table of contents, key concepts, original title, tests & measures] (368461)  62 assessed.mp. [mp=title, abstract, heading word, table of contents, key concepts, original title, tests & measures] (226641)  63 initial consultation*.mp. [mp=title, abstract, heading word, table of contents, key concepts, original title, tests & measures] (272)  64 first consultation*.mp. [mp=title, abstract, heading word, table of contents, key concepts, original title, tests & measures] (187)  65 new consultation*.mp. [mp=title, abstract, heading word, table of contents, key concepts, original title, tests & measures] (22)  66 61 or 62 or 63 or 64 or 65 (547963)  67 exp Mental Disorders/ (526934)  68 Mental Health/ (52386)  69 Mental Health Services/ (31191)  70 mental health programmes.mp. [mp=title, abstract, heading word, table of contents, key concepts, original title, tests & measures] (72)  71 mental disorder*.mp. [mp=title, abstract, heading word, table of contents, key concepts, original title, tests & measures] (103767)  72 mental health*.mp. [mp=title, abstract, heading word, table of contents, key concepts, original title, tests & measures] (177154)  73 mental health service*.mp. [mp=title, abstract, heading word, table of contents, key concepts, original title, tests & measures] (46724)  74 mental illness*.mp. [mp=title, abstract, heading word, table of contents, key concepts, original title, tests & measures] (38572)  75 mentally ill.mp. [mp=title, abstract, heading word, table of contents, key concepts, original title, tests & measures] (14548)  76 PSYCHIATRY/ (23435)  77 PSYCHOLOGY/ (25374)  78 psych*.mp. [mp=title, abstract, heading word, table of contents, key concepts, original title, tests & measures] (1149974)  79 wellbeing.mp. [mp=title, abstract, heading word, table of contents, key concepts, original title, tests & measures] (9251)  80 well-being.mp. [mp=title, abstract, heading word, table of contents, key concepts, original title, tests & measures] (72273)  81 exp MAJOR DEPRESSION/ (113158)  82 depress*.mp. [mp=title, abstract, heading word, table of contents, key concepts, original title, tests & measures] (301012)  83 exp SCHIZOPHRENIA/ (80749)  84 schizo*.mp. [mp=title, abstract, heading word, table of contents, key concepts, original title, tests & measures] (116415)  85 Bipolar Disorder/ (23799)  86 bipolar.mp. [mp=title, abstract, heading word, table of contents, key concepts, original title, tests & measures] (37244)  87 bi-polar.mp. [mp=title, abstract, heading word, table of contents, key concepts, original title, tests & measures] (149)  88 exp Anxiety Disorders/ (73230)  89 anxiety.mp. [mp=title, abstract, heading word, table of contents, key concepts, original title, tests & measures] (188358)  90 ANXIETY/ (52465)  91 personality disorder*.mp. [mp=title, abstract, heading word, table of contents, key concepts, original title, tests & measures] (38270)  92 exp Personality Disorders/ (31006)  93 phobi*.mp. [mp=title, abstract, heading word, table of contents, key concepts, original title, tests & measures] (17541)  94 PANIC/ (1925)  95 Panic Disorder/ (7311)  96 panic.mp. [mp=title, abstract, heading word, table of contents, key concepts, original title, tests & measures] (15917)  97 emotional disorder*.mp. [mp=title, abstract, heading word, table of contents, key concepts, original title, tests & measures] (4326)  98 exp Emotional Trauma/ (14718)  99 emotional trauma*.mp. [mp=title, abstract, heading word, table of contents, key concepts, original title, tests & measures] (14996)  100 emotional problem*.mp. [mp=title, abstract, heading word, table of contents, key concepts, original title, tests & measures] (5701)  101 self-injur*.mp. [mp=title, abstract, heading word, table of contents, key concepts, original title, tests & measures] (6414)  102 exp Self-Injurious Behavior/ (4638)  103 self-harm*.mp. [mp=title, abstract, heading word, table of contents, key concepts, original title, tests & measures] (4974)  104 selfinjur*.mp. [mp=title, abstract, heading word, table of contents, key concepts, original title, tests & measures] (16)  105 selfharm*.mp. [mp=title, abstract, heading word, table of contents, key concepts, original title, tests & measures] (15)  106 self-mutilat*.mp. [mp=title, abstract, heading word, table of contents, key concepts, original title, tests & measures] (1766)  107 selfmutilat*.mp. [mp=title, abstract, heading word, table of contents, key concepts, original title, tests & measures] (7)  108 talking therap*.mp. [mp=title, abstract, heading word, table of contents, key concepts, original title, tests & measures] (190)  109 increasing access to psychological therapies.mp. [mp=title, abstract, heading word, table of contents, key concepts, original title, tests & measures] (16)  110 IAPT.mp. [mp=title, abstract, heading word, table of contents, key concepts, original title, tests & measures] (190)  111 cognitive behavioural therap*.mp. [mp=title, abstract, heading word, table of contents, key concepts, original title, tests & measures] (2904)  112 cognitive behavioral therap*.mp. [mp=title, abstract, heading word, table of contents, key concepts, original title, tests & measures] (13217)  113 CBT.mp. [mp=title, abstract, heading word, table of contents, key concepts, original title, tests & measures] (11214)  114 dialectical behaviour therap*.mp. [mp=title, abstract, heading word, table of contents, key concepts, original title, tests & measures] (168)  115 dialectical behavior therap*.mp. [mp=title, abstract, heading word, table of contents, key concepts, original title, tests & measures] (1533)  116 DBT.mp. [mp=title, abstract, heading word, table of contents, key concepts, original title, tests & measures] (1194)  117 counselling.mp. [mp=title, abstract, heading word, table of contents, key concepts, original title, tests & measures] (10228)  118 counseling.mp. [mp=title, abstract, heading word, table of contents, key concepts, original title, tests & measures] (83025)  119 psychotherap*.mp. [mp=title, abstract, heading word, table of contents, key concepts, original title, tests & measures] (159441)  120 psychoanaly*.mp. [mp=title, abstract, heading word, table of contents, key concepts, original title, tests & measures] (91177)  121 psychodynamic.mp. [mp=title, abstract, heading word, table of contents, key concepts, original title, tests & measures] (16995)  122 interpersonal therap*.mp. [mp=title, abstract, heading word, table of contents, key concepts, original title, tests & measures] (712)  123 IPT.mp. [mp=title, abstract, heading word, table of contents, key concepts, original title, tests & measures] (1012)  124 group therap*.mp. [mp=title, abstract, heading word, table of contents, key concepts, original title, tests & measures] (12057)  125 exp PSYCHOTHERAPY/ (195820)  126 Dialectical Behavior Therapy/ (962)  127 COUNSELING/ (20698)  128 or/67-125 (1683417)  129 37 and 60 and 66 and 128 (1295)  130 limit 129 to up=20150101-20170803 (239) |

| **Table S4: Additions to the CASP (2017)** | | | |
| --- | --- | --- | --- |
| **Area** | **Key question** | **Considerations** | **Source** |
| Intersectionalities | Have issues relating to intersectionalities been fully considered? | Were attempts made to obtain a diverse sample of the population in question? | Modified from Rees, Caird, Dickson, Vigurs, 2014 |
|  |  | Have sample characteristics been fully described, including as a minimum sex, age and ethnicity? | Modified from Rees, Caird, Dickson, Vigurs, 2014 |
|  |  | Has diversity of experience been explored in the findings in relation to intersectionalities (e.g. ethnicity etc)? |  |
|  |  | Have issues relating to intersectionalities been explored in the discussion or limitation sections? |  |
| Service user involvement | Have service users been appropriately involved in the research? | Does the study use appropriate data collection methods to ensure findings are grounded in service users' experiences? | Thomas, Sutcliffe, Harden, Oakley, 2003 |
|  |  | Are the methods appropriate to ensure that data analysis is grounded in service user's views? | Thomas, Sutcliffe, Harden, Oakley, 2003 |
|  |  | Are service users actively involved in the design and conduct of the study? | Thomas, Sutcliffe, Harden, Oakley, 2003 |
|  |  | Are service users leading or controlling the study? | (See Rose, Carr, Beresford, 2018) |
|  |  | Are service users involved in data analysis (e.g. coding, member checking, reviewing transcript) | (See Sweeney, Greenwood, Williams, Wykes, Rose, 2018) |
