# Supplementary material for: A systematic review of qualitative studies of adults’ experiences of being assessed for psychological therapies
Source: Health Expect. 2019 Jan 8;22(2):133–48. doi: 10.1111/hex.12844 (PMC6433316; doi:10.1111/hex.12844)
Supplement: Supplementary file 2 [file HEX-22-133-s002.docx]

# Data Supplement Tables S3 and S5 in landscape

| **Table S3: Data extraction form** | | | | | | | | | | | | | | | |
| --- | --- | --- | --- | --- | --- | --- | --- | --- | --- | --- | --- | --- | --- | --- | --- |
| **About** | | | | | | | | | | | | | | | |
| Authors | | Date of review | Publication year | | Country / region | | Title | Type of paper | Research identity | Service user involvement | Brief description | Stated research aims | Research question | Study setting | Study location |
| **Participants** | | | | | | | | | | | | | | | |
| Groups and numbers | | Inclusion criteria | Recruitment | | Sampling | | Difficulties (mental health) | Sex | Age | Sexual Orientation | Socioeconomics | Employment | Ethnicity | Education | Living situation |
| **Participants continued** | | | | | | | | | | | | | | | |
| Marital status | | Religion | Other | |  | |  |  |  |  |  |  |  |  |  |
| **Methods** | | | | | | | | | | | | | | | |
| Design | | Data collction | Data collected by | | Analysis method | |  |  |  |  |  |  |  |  |  |
| **Assessment (ASM)** | | | | | | | | | | | | | | | |
| What is the ASM for? | | Who initiated the ASM? | Who is the assessor? | | What form does the ASM take? | | Location of ASM | Duration of ASM | Time between ASM and research participation | Is trauma discussed? | Outcome | Other |  |  |  |
| **Theoretical orientation** | | | | | | | | | | | | | | | |
| Underlying theoretical models or concepts | |  |  | |  | |  |  |  |  |  |  |  |  |  |
| **Notes** | | | | | | | | | | | | | | | |
| Any other notable information | |  |  | |  | |  |  |  |  |  |  |  |  |  |
| **Table S5 Further exemplar quotes to illustrate review findings** | | | | | | | | | | | | | | | |
| **Phase of the assessment process** | | | **Theme** | | **Further exemplar quotations** | | | | | | | | | | |
| JOURNEY TO THE ASSESSMENT | | | Distress and desperation | | “*I would have tried anything. I did need help*” [31]*.*  “*Because I felt so unhappy, unhappy in myself ...in whatever I did. I just felt so much unhappiness inside, the whole time. I felt at conflict all the time, with myself”* [31]*.*  “*I saw her because my dissociative symptoms were out of control and my ability to function was severely impaired. I was also feeling suicidal and felt responsible for the abuse*” [33]. | | | | | | | | | | |
|  |  |  | Seeking a new approach | | "*[I] used to bumble from one thing to another in the hope that something helped*" [31].  “*It’s a sense of “this or nothing”*’ [32].  *“I wasn't getting anywhere with previous therapy, I knew I needed a new approach”* [33]. | | | | | | | | | | |
|  |  |  | Gatekeeping | | “*I decided I wanted to get into counselling and then ended up in hospital so I couldn’t actually get to a phone to ring…..It just seems a really ridiculous sort of procedure that they only decide on one day a month, you know, between five hours when you can get a slot. And if you miss out on that then you’ve had it then*” [35].  *“Maybe the doctor’s understanding what (IAPT service) itself are actually about and doing, might help the process of them understanding that they’re not actually forwarding you to what they think they are*” [39].  *“Can people refer themselves? Do they have to go through a GP, which can be very degrading because the GPs are not as sympathetic or I believe as well trained as the staff in depression and mental issues. Going to a GP can be degrading. You only need one kind of flippant locum doctor really to send you crashing*” [37]. | | | | | | | | | | |
|  |  |  | A difficult wait | | “*On that day I was very scared, anxious ...I had never talked with anyone*” (about being raped) [31]  “*Even if it’s only a short call you feel that somebody’s interested in you, but if nobody does, you just feel you’re on an anonymous list, maybe it’s been thrown away…*” [35].  “*At the time of my referral my anxiety was pretty bad and I didn’t know what to do. I would have appreciated some interim information regarding temporary services to help me get by until therapy could begin*” [37].  “*What am I going to have to do to prove I’m not well?*” [38]  “*I have been fighting to get treatment for nearly two years now, and am still being shuffled through assessments. The waiting is ridiculous and dangerous considering the nature of mental health issues*” [38].  “*In the first 7 months there was nothing, absolutely nothing . . . not even recognition of being on a waiting list*” [39]. | | | | | | | | | | |
| AT THE ASSESSMENT:  “I wanted to know that I  could share an aspect of myself” | | | Traumatic, cathartic | | “*I was coming out with all sorts of stuff I didn't expect to emotion-wise. I was connecting with vulnerable feelings; upset that I was usually good at holding in ....it was cathartic”* [31].  “*Above all I was surprised by the luxury that opened up to me…the amount of attention that I was given then. And the fact that such an extensive and good report was written, [and] the number of tests that I did then. … It was actually heart-warming that this happened. I found that … [patient becomes emotional] … that was actually not acceptable, that so much attention was going out to me*” [36].  *"And that really was just like, oh my goodness, the light really went off. And it was just kind of like, Wow!‘ He took me out of the small box I was living in and kind of like put it in perspective I guess*" [34]*.* | | | | | | | | | | |
|  |  |  | Opening up, closing down | | *“I mean I tried to be as honest as I could about certain questions or evaluations, but at the same time the whole time I’m thinking, [chuckles] You‘ve got to watch what you say. You have to watch the wording with how you say certain things and not.., I don‘t want to say not be emotional about how you answer, but definitely don‘t want to show extreme signs of anger or suicidal thoughts or anything like that. Not that I had them but I went in with the preconceived notion that I wasn‘t going to overreact… So yea, I was definitely guarded*” [34].  *“You know, thinking, ―Maybe over time some of this can creep out, [laughs] you know”* [34].  *“Because it could be something that if I explained to another person, they would have said 'no you are silly, don't worry about that', but sometimes what other people think is silly, is a very, very big issue for you”* [31]. | | | | | | | | | | |
|  |  |  | Validation, pathologisation | | “I was actually allowed to feel what I felt” [36].  “*I was asked more about myself, about my personal experiences and … yes, how I, myself, really perceived things … in contrast to what I have experienced … before sometimes … that when you give a sketch of your biography you become immediately labeled*“ [36].  “*Yes, also validation, actually … recognition of what I felt … that I am not—not … a crybaby or overacting … that I, that I was actually allowed to feel what I felt*” [36].  *“I was quite pleased that I was being taken notice of and that I was believed in what I was actually saying to people”* [38].  “*it’s almost like being betrayed, it’s a horrible, horrible feeling not feeling listened to, when that’s what you want*” [39].  *"I felt that a feminist would be on my side and not blame me for what happened*" [33]. | | | | | | | | | | |
|  |  |  | Social identity | | “*I felt that the issues I was dealing with were very female issues*” [31].  *“I never found a therapist that totally understood how culture/ethnicity tied into my experience. The one who at least made an attempt was the only one who helped me*” [33]*.*  "*Female. I don't have to consider the possibility that another male will try to abuse me. I relate better to women*" [33]. | | | | | | | | | | |
|  |  |  | Staff impact | | “*She was so warm -- and it really came through on the phone, and it felt like -- I was in such a vulnerable place in the summer -- that I just soaked it up*” [33].  *“She … helped me gain deeper insight into why I felt what I felt*” [36].  “*The lady I saw was friendly and very easy to talk to – she gave me two assessment sessions as she felt that was what was needed. She wrote me a detailed letter and I felt more positive about actually finally getting some help*” [37].  “*it’s insulting and, you know if I was eight, maybe fine. No. I think if I was eight years old I would still be cross*” [39]. | | | | | | | | | | |
|  |  |  | Assessment techniques | | “*The tests indicated things about both certain aspects of vulnerability, and also certain aspects of personal strengths. I actually rather liked it that this more complete picture became available to my therapist*” [36].  “*I liked the questionnaires, how straightforward they are*” [41].  “*the results of the Meyers-Briggs test was just another way to feel limited and I just did not want to hear it*” [34].  “*I felt like I could explain things just as easily as taking the test… I would have been able to have been a little bit more relaxed and trusting about the process had it just been all conversation and we just got to the problems as they came up. I wouldn‘t feel like the test was just like, bam, this is what it is and then not trusting the test*” [34]. | | | | | | | | | | |
| AT THE ASSESSMENT: “*I didn’t know what rights I had*” | | | Information giving and gathering | | “*.. to allay anxiety I think it would have been a bit better to say …exactly what the format was to begin with and not leave it up to the person to find out….I was put in there without any explanation whatsoever really*” [35].  “*Yes well depression can leave you very confused . . . it was all going over my head, no matter how many questions I asked he just seemed to be explaining it in the same way*” [39].  “*I asked her did she have experience working with survivors of child sexual abuse ... I asked her what being a feminist meant to her ... I actually asked her about the feminist part and she said she learned through her own readings and experiences ... I also asked her if she did dream work because I was into Jungian dream work. I asked her how she felt about Jungian psychotherapy. I wanted to make sure she didn't judge me for writing down my dreams. It's kind of interesting, it's like I wanted to know that I could share an aspect of myself*” [33]. | | | | | | | | | | |
|  |  |  | Authority and agency | | “*It‘s like a conversation, you can agree or disagree*” [34].  “*When you finally got in there and feel like you actually might be speaking to somebody then it was very much about ‘oh no we are not going to speak about anything’ . . . she only wanted me to answer her questions*” [39]. | | | | | | | | | | |
| AFTER THE ASSESSMENT | | | Another difficult wait? | | “*if I, as the patient, am willing to put forth the effort to go through the fire (metaphorically speaking), and … the therapist, is willing to help me through it, then there was an excellent chance that I could take something from the sessions … The coming weeks could not come fast enough*” [34].  “*and so I was like, Oooh, if he can do this now I wonder in the coming weeks what else we can get done*” [34]. | | | | | | | | | | |
|  |  |  | Receiving feedback | | “*General impressions is that it’s very, very accurate, it’s a good record of what me and (the psychologist) talked about over both meetings*” [42].  “*The therapist gave me a narrative that fit me completely. … Afterwards she even wrote it in a letter. I had no more questions about myself. I just had to look at the letter*” [36].  “*Well, I like things to be up front. I like to know what your thought process is and if we‘re…, basically I‘m here to get help with my problems and if you have something you are hiding from me, some judgment or whatever, or some feeling… Then it inhibits me from speaking freely*” [34]. | | | | | | | | | | |
|  |  |  | Making choices | | “*I [would like] more of a choice with counsellors… who’s more suitable to work with you. There wasn’t that much information given about that”* [35].  “*I came to the conclusion that that’s the NHS for you, you know, get them through the system. Don’t offer too many choices otherwise… we won’t be able to afford it or something*” [35].  “*There was no choice of therapy, it was CBT*” [37].  *"I interviewed both of them and asked extensive questions about approach. I knew what I didn't want!" [33].* | | | | | | | | | | |
|  |  |  | Assessor-therapist continuity | | “*It was helpful to be told that she wouldn't be the therapist, because I didn't start feeling too comfortable with her. You sort of keep yourself detached*” *[31].* | | | | | | | | | | |
|  |  |  | Not going on to therapy | | “*I’m used to being disappointed*” [35].  “*If you’re having problems you’re not going to attend you’re going to cancel . . . they should have rung me back at some point to say ‘we haven’t heard from you do you still want our help?*” [39].  “*The best one ... that made me think I’m definitely not coming back was ‘ignore your parents’. Now I live with my family and ... I live in a very old fashioned household ... I can’t ignore my own parent, that’s completely and utterly impractical*” [39]. | | | | | | | | | | |
